# Multicenter evaluation of complex urinary diversion for renal transplantation: outcomes of complex surgical solutions

**DOI:** 10.1007/s00345-024-04934-1

**Published:** 2024-04-17

**Authors:** Luka Flegar, Johannes Huber, Juliane Putz, Christian Thomas, Hendrik Apel, Bernd Wullich, Frank Friedersdorff, Guido Fechner, Manuel Ritter, Karoline Kernig, Karl Weigand, Hans Heynemann, Michael Stöckle, Philip Zeuschner, the Working Group Kidney Transplantation of the German Association of Urology, Luka Flegar, Johannes Huber, Juliane Putz, Christian Thomas, Hendrik Apel, Bernd Wullich, Frank Friedersdorff, Manuel Ritter, Karoline Kernig, Karl Weigand, Hans Heynemann, Michael Stöckle, Philip Zeuschner

**Affiliations:** 1 Department of Urology, Philipps-University Marburg, Baldinger Street, 35043 Marburg, Germany; 2 Department of Urology, University Hospital Carl Gustav Carus, Technische Universität Dresden, Dresden, Germany; 3 Department of Urology and Pediatric Urology, University Hospital Erlangen, Friedrich-Alexander-Universität Erlangen-Nürnberg, Erlangen, Germany; 4 Transplant Center Erlangen-Nürnberg, University Hospital Erlangen, Friedrich-Alexander-Universität Erlangen-Nürnberg, Erlangen, Germany; 5 Department of Urology, Charité-Universitätsmedizin Berlin, Corporate Member of Freie Universität Berlin, Humboldt-Universität zu Berlin, Berlin Institute of Health, Berlin, Germany; 6 Department of Urology, University Medical Center Bonn (UKB), Bonn, Germany; 7 Department of Urology, University Rostock, Rostock, Germany; 8 Department of Urology, University Hospital Halle (Saale), Halle (Saale), Germany; 9 Department of Urology and Pediatric Urology, Saarland University, Homburg/Saar, Germany

**Keywords:** Dialysis, Kidney transplantation, Urinary diversion, Renal insufficiency, Ileal conduit

## Abstract

**Purpose:**

An abnormal lower urinary tract poses significant challenges for transplant surgeons. Besides the ureteral anastomosis to an ileal conduit, there are diverse complex reconstructive solutions. Due to its rarity, standardization and teaching of complex urinary diversion is extremely difficult.

**Methods:**

The indications and outcomes of complex urinary diversions after kidney transplantation (KT) were retrospectively investigated at eight urologic transplant centers including a current follow-up.

**Results:**

Of 37 patients with 21 (56%) males, vesicoureteral reflux (24%), spina bifida (22%), and glomerulonephritis (12%) were the most common causes of terminal renal failure. In 30 (81%) patients, urinary diversion was performed before KT, at a median of 107.5 (range, 10; 545) months before. Transplantations were held at a median patient age of 43 (10; 68) years, including six (16%) living donations. Urinary diversion was modified during 12 (32%) transplantations. After KT, the ileal conduit was the most common incontinent urinary diversion in 25 (67%) patients; a Mainz pouch I and bladder augmentation were the most frequent continent diversions (each n = 3). At a median follow-up of 120 months (range 0; 444), 12 (32%) patients had a graft failure with a 5-year graft survival of 79% (95%CI 61; 90). The median overall survival was 227 months (168; 286) and the 5-year overall survival 89% (69.3; 96.4).

**Conclusion:**

The mid-term kidney transplant function with complex urinary diversion appears to be comparable to transplants with regular urinary diversions. Hence, complex urinary diversion should always be considered as a surgical option, even during transplantation, if necessary.

**Supplementary Information:**

The online version contains supplementary material available at 10.1007/s00345-024-04934-1.

## Introduction

Renal transplantation was first successfully performed in Boston in 1954. It represents the preferred and most effective treatment option for patients with end-stage renal disease (ESRD) [1]. Approximately 6% of kidney transplant patients experience terminal renal insufficiency due to congenital abnormalities, such as urethral valves, vesicoureteral reflux, neurogenic bladder disorders, prune-belly syndrome or other rare syndromes [2, 3]. These anomalies account for a quarter of all terminal renal insufficiencies in pediatric patients and 15% in adults [4].

The treatment of these patients with anatomical or functional lower urinary lower tract dysfunction poses great challenges for renal transplant surgeons and requires careful consideration. On the one hand, congenital or acquired urological diseases not only contribute to native kidney failure but on the other hand can also adversely affect the outcome of renal transplantation. Therefore, restoring the normal function of the lower urinary tract is crucial. Advancements in lower urinary tract evaluation and management, including medical therapy and intermittent self-catheterization (ISC), alongside surgical procedures, can achieve this objective [5].

In this regard, a unique surgical milestone occurred in 1966, when Kelly and colleagues reported the first series of successful kidney transplantations in seven patients with ileal urinary diversion [6]. Before, having an intact natural lower urinary tract was considered essential for transplant eligibility to avoid infections or urosepsis in immunocompromised patients and to protect the renal allograft [7]. After the first report on the successful implantation of a kidney transplant into a colon conduit by Tunner et al. in 1971, the indications for complex urinary diversions in kidney transplantation have been continuously widened [8]. Today, many surgical options before or even during kidney transplantation (KT) appear to be viable solutions for complex situations in patients with urinary tract anomalies, including bladder augmentation, continent or incontinent urinary diversions using intestinal segments [9, 10].

However, data is scarce about the outcomes of these complex urinary diversions after KT. Therefore, we aimed to investigate their outcomes at eight urologic transplant centers, with a special focus on renal allograft function and patient survival, including a current follow-up.

## Materials and methods

### Patient cohort

This multi-institutional retrospective study included all patients with a KT and a complex urinary diversion from eight German tertiary referral centers. Complex urinary diversion was defined as the existence of a continent (Mitrofanoff appendicovesicostomy, neobladder, pouch) or incontinent (ileal or colon conduit, ureterocutaneostomy) urinary diversion with the graft attached to it.

Patient demographics including the underlying uro- and nephropathy were obtained, as well as the individual surgical history and urinary diversion before kidney transplantation. Regarding KT, its type, date and specifics were obtained. For each patient, a recent follow-up was conducted, including serum creatinine and glomerular filtration rate one, two, five- and ten-years post KT. Patient and death censored graft survival were estimated.

### Statistical analysis and ethics statement

Categorical variables were reported as frequencies and proportions, continuous data as the median and range. Survival analyses were performed with the Kaplan Meier estimation method and were stratified by the timing of urinary diversion (before vs. after KT), the type of KT (living vs. brain dead donor) and type of urinary diversion (continent vs. incontinent); the respective groups were compared with log-rank tests. The statistical analyses were performed with SPSS version 25 (IBM, Armonk, USA). This study was conducted according to the Declaration of Helsinki and approved by the local responsible ethical review boards (reference 106–18).

## Results

Of 37 patients with complex urinary diversions at eight German tertiary referral centers, 21 (57%) were male. Median BMI of all patients was 24.5 kg/m^2^ (range 16.0; 36.2) (Table 1). The most common causes of kidney insufficiency were vesicoureteral reflux (24%), spina bifida (22%) and glomerulonephritis (11%). In 30 (81%) patients, a urinary diversion had already been performed before kidney transplantation at a median patient age of 29 years (range 0; 62), at a median of 107.5 months (10; 545) earlier. The most common urinary diversion before KT was an ileal conduit (48.6%). Patients who had received urinary diversion before transplantation were less likely to have a history of urothelial carcinoma, were more likely to be female, and had fewer intraoperative complications. The groups were otherwise comparable (Table S1 in online supplement).

**Table 1:** Patient characteristics of the analyzed cohort

n = 37	
Age	43 (10; 68) years
Sex	21 (56.8%) male
BMI	24.5 kg/m^2^ (range 16.0; 36.2)
Donor	6 (16.2%) living kidney donations
Urinary diversion prior to KT performed	30 (81.1%)
Most common urinary diversion prior to KT	ileal conduit (48.6%)
Modification of urinary diversion during KT	12 (32.4%)
Continent urinary diversion after KT	8 (21.6%)
Intraoperative complications	2 (5.4%)
Postoperative complications	7 (18.9%)
Serum creatinine at 2-years	1.5 mg/dl (range 0.5; 9.95)
Serum creatinine at 5-years	1.6 mg/dl (range 0.7; 10.1)
Graft failure	12 (32.4%)
Most common cause for graft failure	6 (50%) chronic rejections

**Fig. 1:**
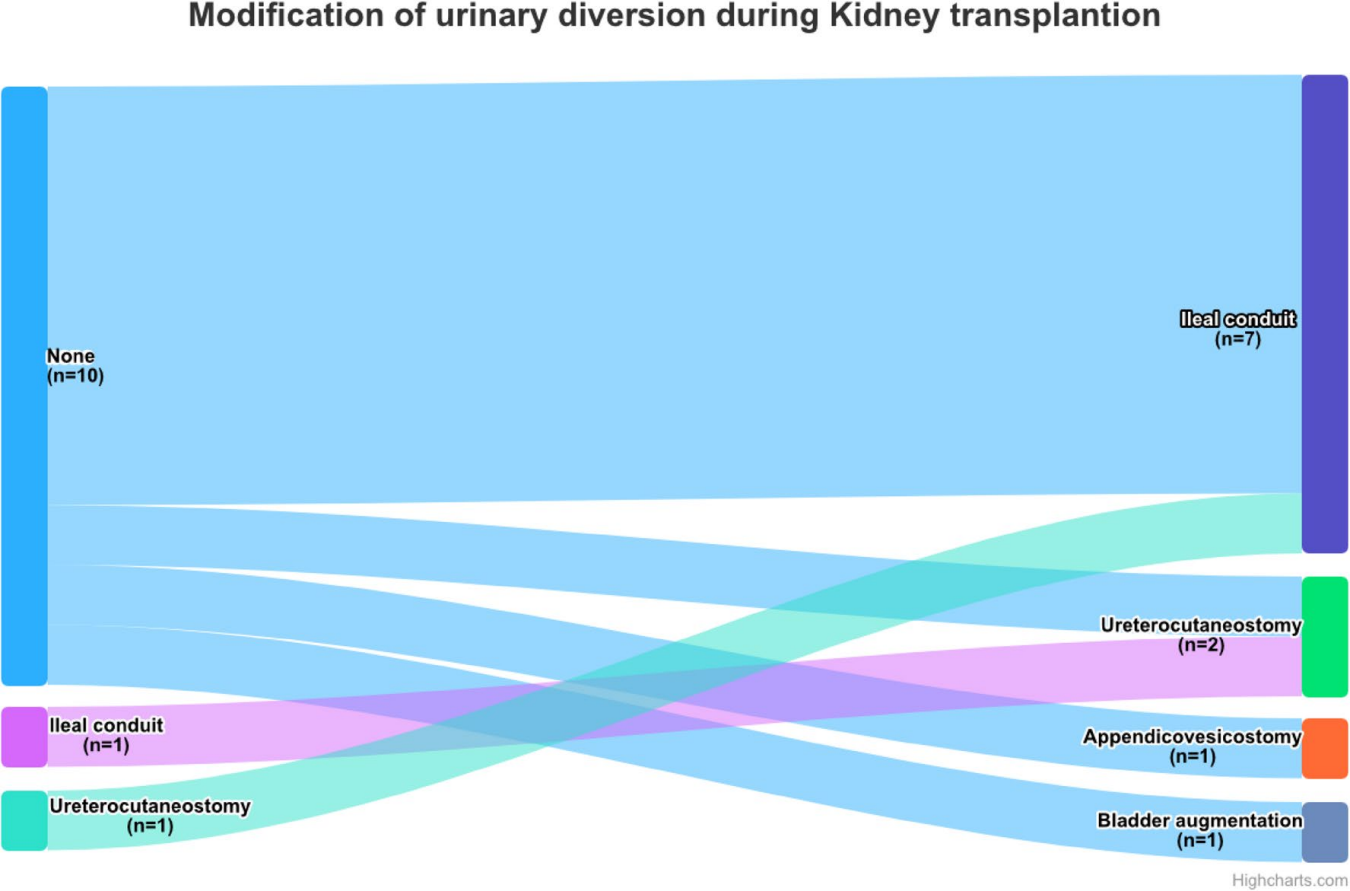
Sankey diagram illustrating how the urinary diversions were modified during KT

The kidney transplantations were carried out between 1986 and 2023 at a median patient age of 43 years (range 10; 68), six (16.2%) KT were living kidney donations. During 12 (32.4%) KT, the urinary diversion was modified (see Fig. 1 precisely illustrating the modification of the urinary diversions during KT). One (3%) patient had a pre-existing ureterocutaneostomy which was changed to an ileal conduit, in another patient with an ileal conduit a ureterocutaneostomy was performed. One KT was held with a simultaneous bladder augmentation; in another KT, a Mitrofanoff appendicovesicostomy was created. In all other KT with simultaneous urinary diversion, ileal conduits were performed. Intraoperative complications (one bowel perforation and one urinary bladder perforation) occurred in two (5%) patients, while postoperative complications (Clavien-Dindo 3b in four patients: spleen rupture, anastomotic insufficiency, wound dehiscence, perirenal hematoma) were reported in seven (19%) patients.

After KT, 8 (22%) patients had a continent urinary diversion, 3 (8%) had a Ileocecal (Mainz) Pouch, 3 (8%) a bladder augmentation, 1 (3%) a Mitrofanoff appendicovesicostomy and 1 (3%) an orthotopic neobladder. As incontinent urinary diversion, most patients had an ileal conduit (68%), 2 (5%) also a ureterocutaneostomy, and 2 (5%) a colon conduit.

### Follow-up

**Fig. 2:**
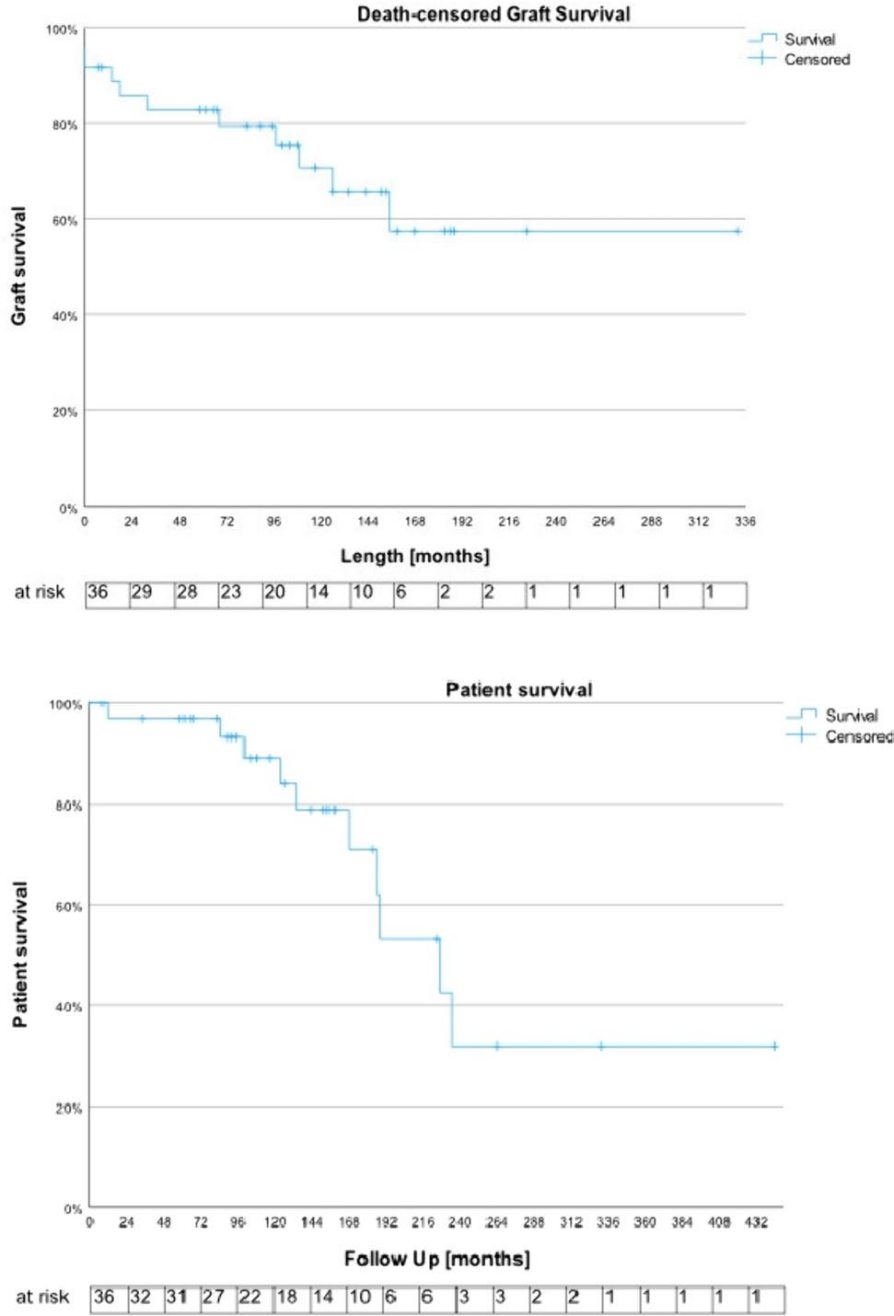
Death censored graft and patient survival of the analyzed cohort; the number of the respective patients at risk is indicated below

At a median follow up of 120 months (range 0; 444), the serum creatinine was 1.5 mg/dl (range 0.5; 9.95) two years and 1.6 mg/dl (0.7; 10.1) five years post KT, the GFR was 46.6 ml/min/m^2^ (27.9; 90) and 44 (30; 83), respectively (Figure S1). Twelve (32.4%) patients had a graft failure at a median of 32 (0; 155) months after KT. The most common causes were chronic rejections in 6 (50%) cases, two were related with recurrent urinary tract infections. The median death censored graft survival was not reached, and the mean graft survival was 222.9 months (95%CI 170.8, 275.1) with a 1-year, 3- and 5-year death censored graft survival of 88.7% (95%CI 72.6; 95.6), 82.8% (65.6; 91.9) and 79.3% (61.3; 89.6), respectively (Fig. 2, Table 1). Two (5.4%) grafts were explanted at a median of 120 (83; 157) months after KT. The graft survival was neither impacted by the timing of urinary diversion (during vs. before KT, p = 0.2), nor by the type of urinary diversion (incontinent vs. continent, p = 0.2, Fig. 2). None of the patients receiving a living kidney donation had a graft loss; however, their graft survival was not significantly longer compared to dead donors (p = 0.1).

**Fig. 3:**
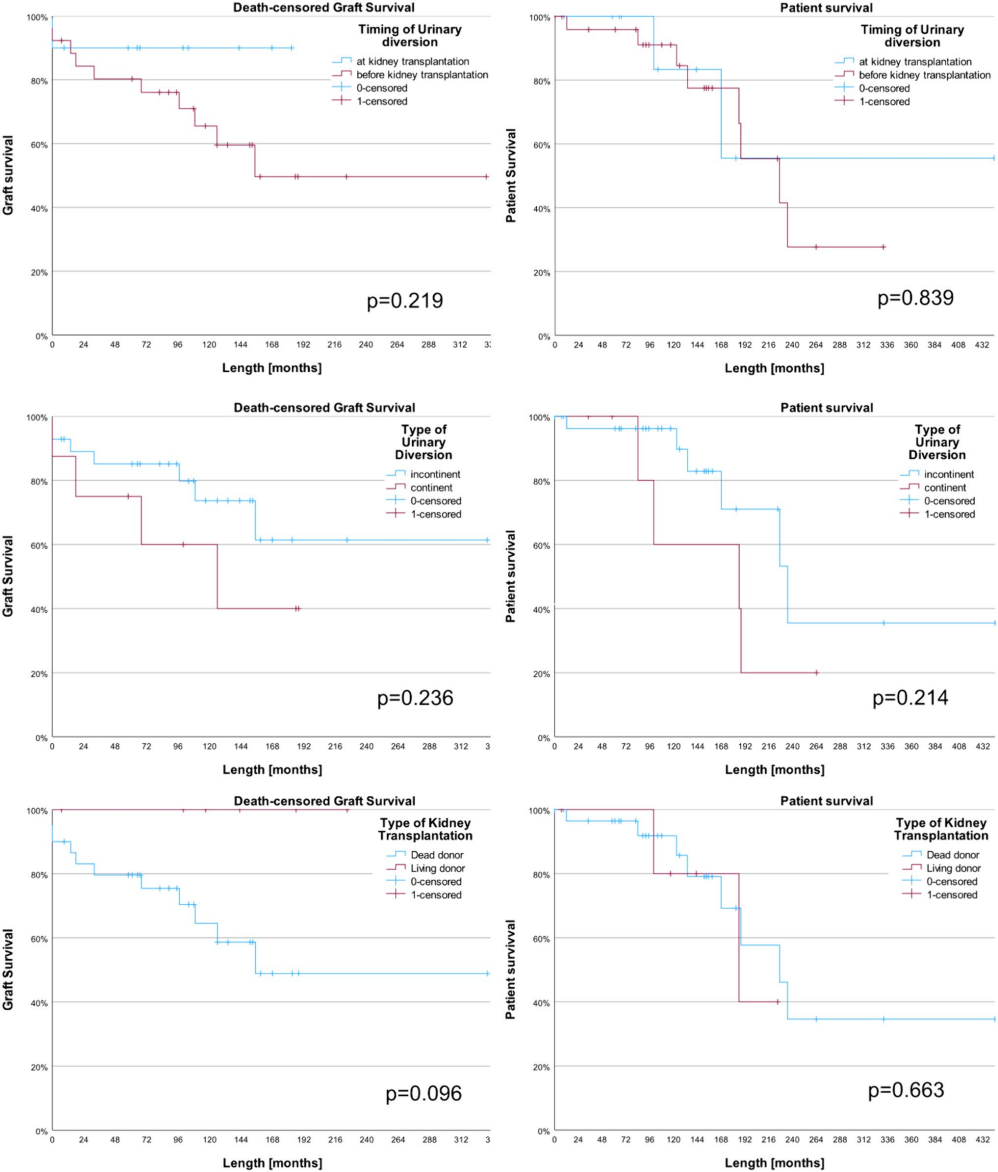
Death censored graft survival (left column) and patient survival (right column), stratified by timing of urinary diversion (first row, before vs. during kidney transplantation), type of urinary diversion (second row, incontinent vs. continent urinary diversion) and type of kidney transplantation (third row, dead vs. living donor)

Within the follow up period, 10 (27%) patients have died and the median patient survival was 227 (95%CI 167.6; 286.4) months with a 1-year, 3- and 5-year overall survival of 96.7% (95%CI 80.4; 100), 93.2% (75.3; 98.2) and 89% (69.3; 96.4%), respectively (Table S2 in online supplement). The patient survival was neither impacted by the timing nor type of urinary diversion or the type of kidney transplantation (Fig. 3).

## Discussion

The anatomically or functionally abnormal urinary tract poses significant challenges even to experienced kidney transplant surgeons. On the one hand, an abnormal urinary tract in kidney transplant recipients is a rare situation, as approximately only 1% of kidney transplantations are performed into patients with (prior) supravesical diversion or bladder augmentation. On the other hand, a kidney transplantation itself may be a complex surgical intervention [11–13]. Therefore, data is scarce about the mid- and long-term follow-up of kidney transplant patients with complex urinary diversion. In the present study, we investigated the outcomes of in total 37 kidney transplant patients with complex urinary diversion at eight urologic departments. In brief, most urological diversions were performed before surgery, and did not affect the mid- and long-term outcomes, which were comparable with those of transplants recipients with regular urinary diversions. This might be related with the fact, that all patients had their surgeries in urological departments exclusively.

In our cohort, the median age of the kidney recipients at the time of KT was 43 years, making them younger than the general recipients. Wolfe et al. reported that in the US, almost half of the patients awaiting renal transplants are older than 50 years [14]. While kidney transplantation can still be safely performed in older age groups, elderly recipients have shown to experience significantly lower graft and patient survival rates, along with a significantly higher risk of graft loss and patient mortality [15]. Furthermore, advancing age has also been associated with longer dialysis time. Aufhauser et al. demonstrated that patients with a pre-transplant dialysis time of ≥ 10 years had worse outcomes compared to those who were transplanted preemptively or with shorter time on dialysis [16]. This younger patient age in our cohort compared to most kidney transplantations can be explained with their underlying diseases, as one out of four patients in the present analysis had ESRD due to vesicoureteral reflux (VUR). In line with these results, Mattoo described VUR as the commonest congenital urinary tract abnormalities in childhood, which is diagnosed mostly after an episode of urinary tract infection (UTI) and is seen in 5.2% of transplanted patients and 3.5% of dialysis patients [17].

From this point of view, it is very encouraging that the functional results of these 37 patients were not only in line with published data, as the 5-year graft survival in the literature ranges between 65 and 75% [7, 12, 18–21]. The 5-year death censored graft survival rates within this cohort of 79.3% appeared to be even slightly superior to published data. This might be related with new immunosuppressive regimens, for instance, as some of the published cohorts are already 20 years old. However, the first kidney transplantation comprised within our cohort dated back to 1986 and the latest was performed in 2023. Moreover, according to recent analysis of the German Organ Procurement Organization from 2019, the 3-year overall survival of kidney transplant patients was 91.8% (95% CI 90.4%; 93.1%), and the graft survival 90.6% (89.1%; 91.9%), compared to an OS of 93.2% and graft survival of 83% within this cohort. Potentially, these excellent results might be related with a tight and well-organized urological follow-up.

Another important aspect to consider when analyzing the graft survival in patients with complex urinary diversions undergoing kidney transplantation surely is the timing of urinary diversion. In general, the urinary diversion can be performed (1) before, (2) simultaneously or (3) after kidney transplantation. Most studies recommend surgery for urinary diversion prior to KT since the absence of steroids and immunosuppressants enable improved wound healing [2, 20]. Consistent with these recommendations, 30 (81.1%) patients in our cohort received their urinary diversion before KT, at a median of 107.5 months (10; 545) earlier. Moreover, their graft survival was not impacted by the timing of kidney transplantation. However, other studies argue that urinary diversion after KT is associated with a lower risk of damaging important structures [2, 22]. It may even be considered necessary in some cases, such as when dealing with newly diagnosed bladder cancer due to immunosuppression [23]. In our cohort, intraoperative complications occurred only in two patients and therefore synchronous change of urinary diversions during KT seems also feasible.

Apart from the variations in timing for urinary diversion, we also observed a broad range of continent and incontinent urinary diversions in our patient cohort. While the ileal conduit was the most commonly performed urinary diversion in 25 patients (67%), there were also several other techniques included in our study cohort: a Mainz Pouch (8.1%), bladder augmentation (8.1%), a Mitrofanoff appendicovesicostomy (2.7%), and and orthotopic neobladder (2.7%). Additionally, one patient with a pre-existing ureterocutaneostomy received an ileal conduit during KT, while another patient with an ileal conduit obtained an ureterocutaneostomy. Despite the heterogeneity of these surgical approaches, they were found to be challenging yet technically feasible, with excellent and encouraging outcomes. In line with these results, Chaykovska et al. described a further urinary diversion with an ureteroureterostomy between the transplant and native ureter, which yielded favorable functional results [24].

Therefore, one has to note that our study clearly demonstrates that complex urinary diversions in renal transplantation, as described above, are generally feasible due to urological involvement in KT. However, there is a current trend among many transplant surgeons to argue that they do not necessarily need to be experienced in urologic urinary diversions. As a consequence, the role of urology in KT has significantly declined on an international level over the past decade [25, 26]. Nonetheless, it is essential to recognize that for patients with complex urological reconstructions, having urologists familiar with the anatomy and experienced in urological surgery can be highly advantageous and lead to optimal quality of care for this special patient group [25]. Urologists should also be integral members of a multidisciplinary team to participate in the initial evaluation and selection of potential recipients, as well as in postoperative care. This is particularly important since renal transplant recipients are often at increased risk of conditions such as benign prostatic hypertrophy (BPH), voiding dysfunction, and various other urological allograft-related complications [26–28].

While the current study provides valuable observations regarding complex urinary diversion for renal transplantation, it is essential to acknowledge its inherent limitations and to interpret the findings within the context of its retrospective, multi-institutional design. Additionally, the analysis was based on a small sample size, which should be taken into consideration when interpreting the results.

To conclude, the absence of an intact lower urinary tract should clearly not be considered as a contraindication to a (successful) kidney transplant. Our data clearly demonstrates that complex urinary diversion allows a stable kidney function which is almost comparable to transplants with regular urinary diversions. Hence, complex urinary diversion should always be considered as a surgical option, even during transplantation, if necessary. In this special situation, a broad expertise in urological diversions appears to be not only helpful, but rather a prerequisite, wherefore at least in these situations a urological standby should be organized during surgery.

## Supplementary Information

Below is the link to the electronic supplementary material.

Supplementary file1 (DOCX 111 KB)

## Abbreviations

BPH: Benign prostatic hypertrophy
DT: Dialysis time
ESRD: End-stage renal disease
ISC: Intermittent self-catheterization
KT: Kidney transplantation
VUR: Vesicoureteral reflux
