# Supplementary material for: Multicenter evaluation of complex urinary diversion for renal transplantation: outcomes of complex surgical solutions
Source: World J Urol. 2024 Apr 17;42(1):239. doi: 10.1007/s00345-024-04934-1 (PMC11023965; doi:10.1007/s00345-024-04934-1)
Supplement: Supplementary file 1 — Supplementary file1 (DOCX 111 KB) [file 345_2024_4934_MOESM1_ESM.docx]

# Supplementary Material

## Figure S1.


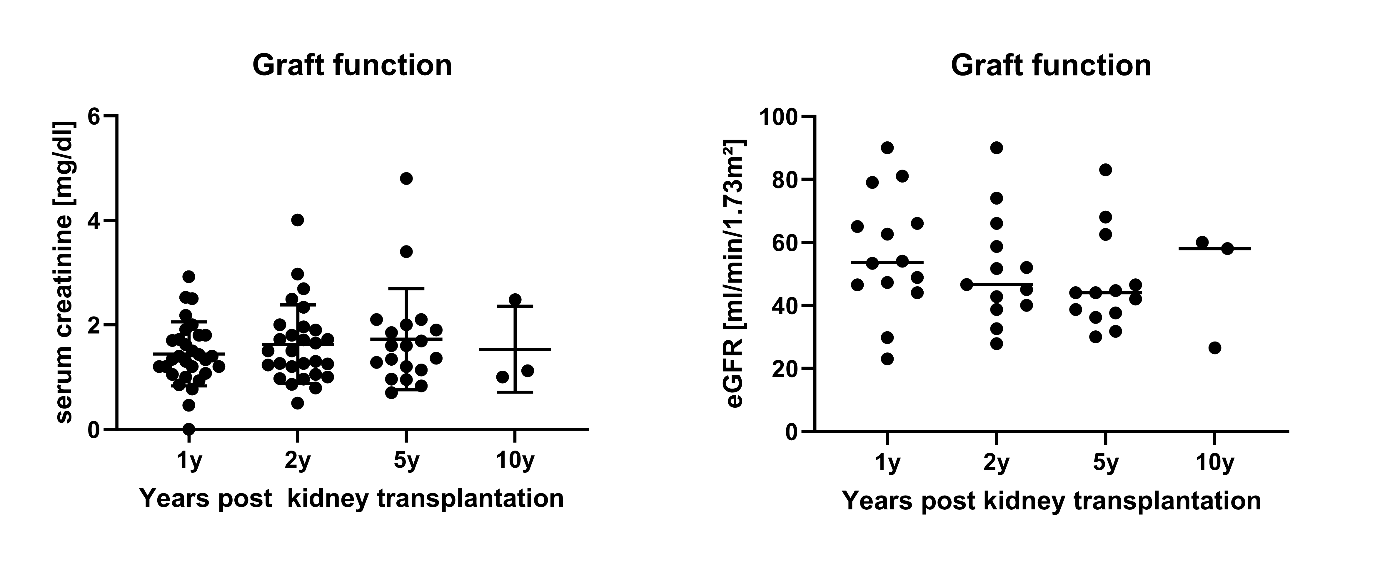


**Figure S1**. Follow-Up of graft function 1, 2, 5 and 10 years after kidney transplantation. Each dot represents one patient; mean an standard deviation are additionally indicated.

## Table S1. Comparison between patients with an existing urinary diversion prior to kidney transplantation and those who had it modified during KT.

|  | **Pre-existing urinary diversion (n=25)** | **Modification of urinary diversion during KT (n=12)** | **p-value** |
| --- | --- | --- | --- |
| main disease  spina bifida  glomerulonephritis  urothelial CA  reflux | 7 (28%)  3 (12%)  0 (0%)  8 (32%) | 1 (8.3%)  1 (8.3%)  4 (33.3%)  1 (8.3%) | 0.232  1.0  **0.007**  0.220 |
| age at urinary diversion | 29.5 (0; 62) | n.a. | n.a. |
| age at TX | 41 (10; 68) | 46 (17; 68) | 0.203 |
| sex male | 11 (44%) | 10 (83.3%) | **0.035** |
| BMI | 24.1 (19.4; 36.2) | 25.5 (16; 34.6) | 0.865 |
| living donation | 4 (16%) | 2 (16.7%) | 1.0 |
| intraoperative complication | 1 (4%) | 1 (8.3%) | **0.048** |
| Serum creatinine at 2-years | 1.65 (0.6; 9.95) | 1.5 (1; 2.34) | 0.946 |
| Serum creatinine at 5-years | 1.5 (1.48 (0.7; 10.09) | 1.65 (0.83; 2.1) | 0.970 |
| Graft failure | 11 (44%) | 1 (8.3%) | 0.058 |

## Table S2. One, three, five and ten years overall and death censored graft survival including 95% confidence intervals.

|  | **Overall survival** | **Death censored graft survival** |
| --- | --- | --- |
| 1 year | 96.7% (80.4; 100) | 88.7% (72.6; 95.6) |
| 3 years | 93.2% (75.3; 98.2) | 82.8% (65.6; 91.9) |
| 5 years | 89% (69.3; 96.4) | 79.3% (61.3; 89.6) |
| 10 years | 84.1% (62.2; 93.8) | 65.6% (44.3; 80.4) |
